# Long-term outcomes of coracoid bone block osteolysis: case report

**DOI:** 10.11604/pamj.2022.42.274.36403

**Published:** 2022-08-12

**Authors:** Arthur Floquet, Steven Roulet, Luc Favard

**Affiliations:** 1 Department of Orthopedic Surgery, La Milétrie University Hospital, Medical University of Poitiers, Poitiers, France,; 2 Elsan, Belledonne Clinic, Gabriel Péri, St-Martin-d´Hères, France,; 3 Department of Orthopedic Surgery, Trousseau University Hospital, Medical University François Rabelais of Tours, Tours, France

**Keywords:** Latarjet procedure, complication, osteolysis, case report

## Abstract

Latarjet procedure is reliable and reproductible procedure for treatment of anterior shoulder instability. Results are durable but complications can occur: neurological injury, non-union, infection, stiffness, osteolysis of coracoid bone block, recurrence of dislocation, osteoarthritis. We present the case of two patients with complication not described in the literature: osteolysis of the neck of the scapula around screws discovered at 8 and 20 years after initial surgery. These patients presented with previous pain which motivated consultation. Imaging showed coracoid bone block osteolysis, extensive geode weakening articular surface of scapular glenoid, around screws which are not bi-cortical. Screws were removed, osteolysis was filled with iliac cancellous bone graft and joint capsule was re-tensioned. Results of this procedure at one year were clinical and radiological satisfactory.

## Introduction

Latarjet procedure [1] is reliable and reproducible procedure for treatment of anterior shoulder instability [2]. Short-term and long-term results are satisfactory [3-6]. Early complications are well described in the literature [7-10] such as neurological damage, pseudarthrosis, infection, malposition of the graft, stiffness. Late complications are also known: conflict pain with screws [11], osteolysis of the coracoid [12-14], recurrence of instability, osteoarthritis [15]. However, no case of scapular neck osteolysis as important around screws is described in literature.

In this article, we report the case of 2 patients with this complication discovered at 8 and 20 years post-operatively, clinical and radiographic results one year after hardware removal, curettage and filling bone defect.

## Patient and observation

### Clinical findings

**Case 1:** this was a 28-year-old male, no medical history, right handed, property agent. He underwent right shoulder Latarjet procedure 8 years previously with osteosynthesis by 2 semi-threaded malleolar screws. Since 2 years, he presented anterior pains which appeared initially during the practice of golf, then progressively during the activities of daily life. No trauma, dislocation or sub-dislocation was reported. Active and passive ranges were identical: antepulsion 170°, abduction 170°, external rotation 180°, internal rotation T10. No apprehension or hyperlaxity was found during the clinical examination. Testing of rotator cuff was unremarkable. Subjective shoulder value (SSV) score was 90 and constant score was 93.

**Case 2:** this was a 44-year-old male, no medical history, right-handed, computer engineer with. He underwent right shoulder Latarjet procedure 20 years previously with osteosynthesis by a semi-threaded malleolar screw. Since 2 years, he presented anterior pains. They initially occurred during physical activity (ping-pong and pétanque), then progressively during activities of daily life. No trauma, dislocation or sub dislocation was reported by the patient. Active and passive ranges were identical: antepulsion 180°, abduction 160°, external rotation 150°, internal rotation T10. Apprehension and hyperlaxity were evident on clinical examination. Gagey test and recentring test were positive, but there was no furrow sign. Testing of rotator cuff was unremarkable. SSV score was 70 and constant score was 90.

### Diagnosis assessment

**Figure 1:**
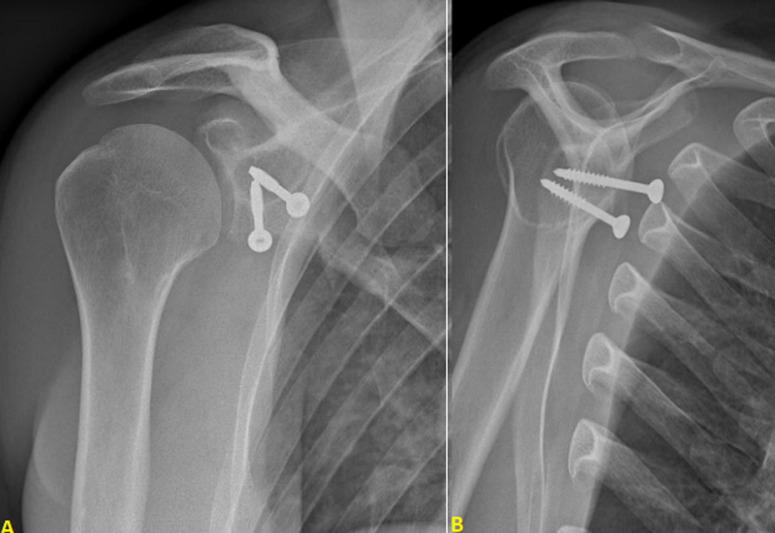
(A,B) frontal and lateral X-rays of right shoulder showing a geode in scapular neck

**Figure 2:**
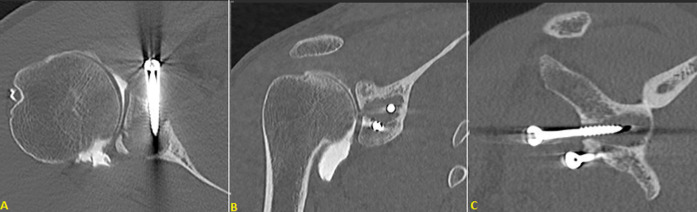
(A,B,C) CT scan of right shoulder; axial, coronal and sagittal slices, joint communication

**Case 1:** X-rays showed osteolysis around proximal screw which was not bi-cortical. There was no geode around distal screw which was bi-cortical. There was no sign of omarthrosis (Figure 1). Computed tomography (CT) scan showed complete osteolysis of the coracoid bone block. Major geode was visible around proximal screw flush with glenoid articular surface communicating with joint through a thin opening. Volume of the bony defect was 8.3 cm^3^ (Figure 2).

**Figure 3:**
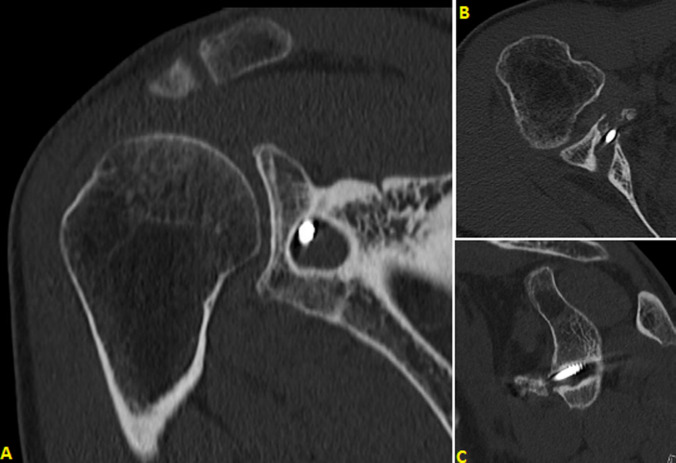
(A,B,C) CT scan of right shoulder; axial, coronal and sagittal slices

**Case 2:** X-rays showed osteolysis around screw which was not bi-cortical. There was no sign of degenerative arthroplasty. Ultrasound was performed and showed no rotator cuff damage. CT scan revealed complete lysis of coracoid bone block. There was a geode around screw flush with glenoid articular surface. The volume of the bone defect was 2.2 cm^3^ (Figure 3).

**Therapeutic intervention:** for both patients, surgical steps were identical. Initial deltopectoral approach was repeated, scapular neck anterior surface was first exposed by performing a subscapular decision and then a horizontal arthrotomy. Screws were removed without difficulty. An intra-articular check was carried out: no effraction was found but soft cartilaginous zone was palpated probably corresponding to place where geodes were in contact with cartilage. Screw hole was then slightly enlarged in order to curettage soft tissue of geode and fill it with cancellous bone taken from the iliac crest. Cavity was then closed by impacting an iliac cortical graft. Finally, joint capsule was tensioned to 45° of external rotation using 2 anchors at anterior edge of glenoid as for a Bankart technique [16]. Patients were instructed post-operatively to immobilise their shoulder for 15 days with an elbow to body and then to perform self-rehabilitation exercises [6].

### Follow-up and outcomes

**Figure 4:**
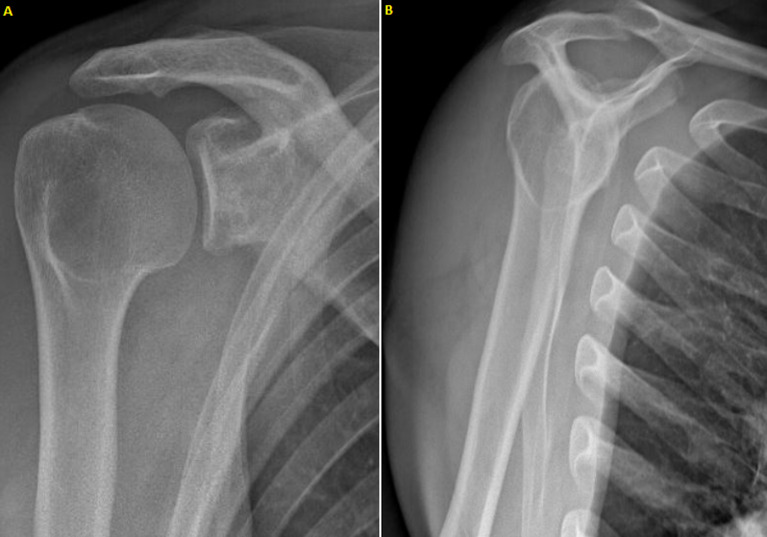
(A,B) X-rays at 12 months post-operatively (lamy side and front)

**Case 1:** patient was reassessed 12 months after surgery, he was pain free, physical activities could be resumed without restriction at same level as before the onset of symptoms. Active range of motion was identical to passive range of motion: antepulsion 180°, abduction 170°, external rotation 180°, external rotation 290°, internal rotation T8. No apprehension or hyperlaxity was detected during the clinical examination or on questioning. Constant score was 99. SSV score was 95. X-rays documented integration of the bone graft into scapular neck (Figure 4).

**Figure 5:**
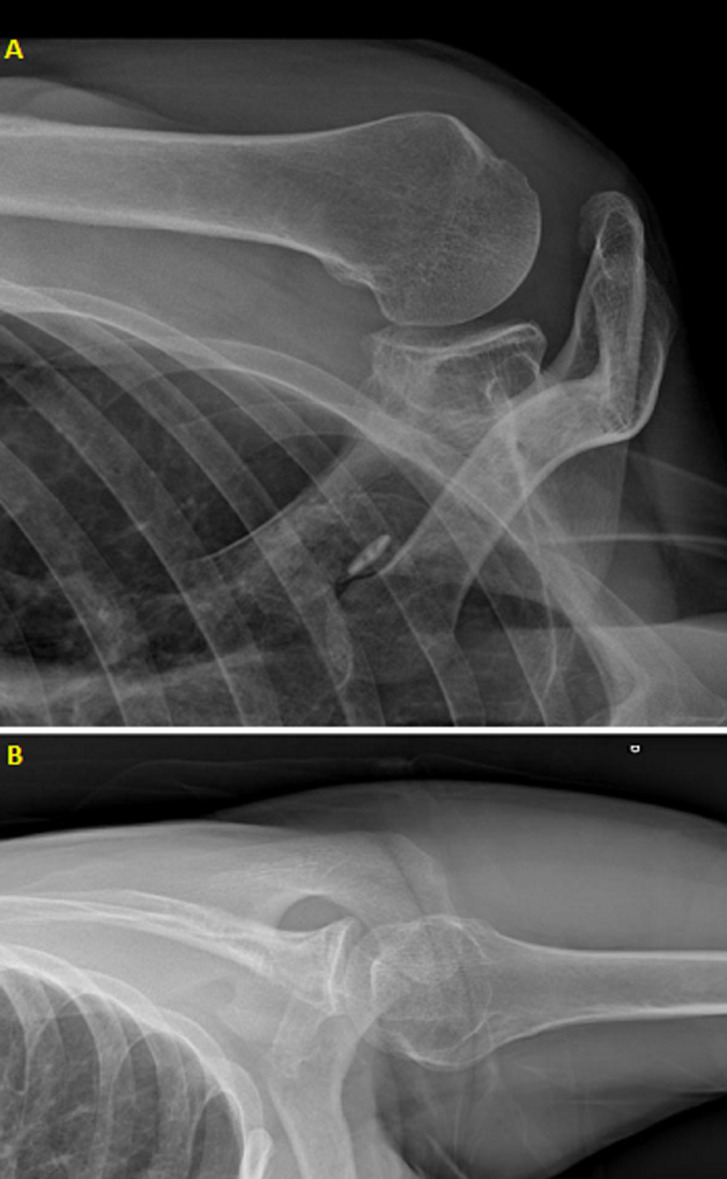
(A,B) X-rays at 13 months after surgery (front and axillary side)

**Case 2:** patient was reassessed 13 months after surgery, he was pain free, physical activities could be resumed without restriction at same level as before the onset of symptoms. Active range of motion was same as passive: antepulsion 180°, abduction 170°, external rotation 140°, internal rotation T7. No apprehension or hyperlaxity was evident during clinical examination or on questioning. Constant score was 100. SSV score was 95. X-rays documented integration of bone graft into scapular neck (Figure 5).

**Patient perspective:** these 2 patients consider themselves healed. They are satisfied with complete medical and surgical management.

**Informed consent:** these patients were informed about the publication of this case series, why their case was special and the authors' intention to publish their case.

**Patients consent:** patients gave their consent for their images and other clinical information to be reported in the journal.

## Discussion

Scapular neck osteolysis around osteosynthesis screws after Latarjet procedure is a long-term complication that rare because it has not been reported in literature. The main reason for consultation is pain. Hardware removal, osteolysis filling and capsular reattachment provide short-term relief and prevent occurrence articular fracture of scapular glenoid.

A few cases of scapular glenoid osteolysis have been described but with use bio-absorbable screws [17]. These osteolysis are limited and correspond at space left free by resorption of material. Images of osteolysis around osteosynthesis screws can be seen on X-rays of some articles but remain less significant than in our patients and therefore did not require specific management [18].

Pain was only symptom in these two patients and occurred with an interval of 8 and 20 years. In case of pain due to conflict between subscapularis muscle and screw heads, discomfort appeared earlier, on average at 29 months [11].

In both patients, pre-operative imaging revealed large geodes under articular surface, around screws which was not bi-cortical. Mechanical weakness of subchondral bone could also contribute to pain phenomena in addition with possible pain on screws head. Bone grafting of this geodes appears to be essential in these cases to avoid a joint fracture in long term.

Explanation for scapular neck osteolysis is probably mechanical, due to micro-mobility of the screws which was not bi-cortical, after lysis or non-union coracoid bone blocks. Osteosynthesis with mono-cortical and/or cannulated screws increases the risk of non-union [18]. Lysis of coracoid bone block is possible with any type of fixation, but bi-cortical screw fixation increases compression on coracoid bone blocks and thus risk of osteolysis [19].

This rare complication would only occur after lysis or non-union coracoid bone block but fixed by one or more mono-cortical screws. Thus, fixation with bi-cortical screws or with cortical buttons [20] would avoid this complication.

## Conclusion

Long-term pain after Latarjet procedure should be investigated for research osteolysis of scapular neck after mono-cortical fixation of coracoid bone block. In absence of osteoarthritis signs and others abnormalities on standard X-rays, further investigations should be carried out with CT scan. Hardware removal, filling geode with cancellous bone graft and capsular retensioning are likely to provide short-term relief and prevent occurrence articular fracture of scapular glenoid.
